# Supplementary material for: Pyruvate Dehydrogenase Kinase 1 inhibition mediated oxidative phosphorylation enhancement in cartilage promotes osteoarthritis progression
Source: BMC Musculoskelet Disord. 2023 Jul 20;24:597. doi: 10.1186/s12891-023-06585-6 (PMC10357736; doi:10.1186/s12891-023-06585-6)
Supplement: Supplementary file 1 — Additional file 1. [file 12891_2023_6585_MOESM1_ESM.zip › MF.Gsea.1653627316242/GOMF_S_ADENOSYL_L_METHIONINE_BINDING.html]

Details for gene set GOMF\_S\_ADENOSYL\_L\_METHIONINE\_BINDING[GSEA]

|  || Dataset | 55457\_collapsed\_to\_symbols.cls.cls #OA\_versus\_normal.cls.cls #OA\_versus\_normal\_repos |
| Phenotype | cls.cls#OA\_versus\_normal\_repos |
| Upregulated in class | OA |
| GeneSet | GOMF\_S\_ADENOSYL\_L\_METHIONINE\_BINDING |
| Enrichment Score (ES) | 0.45069063 |
| Normalized Enrichment Score (NES) | 1.3095767 |
| Nominal p-value | 0.16018307 |
| FDR q-value | 0.40257087 |
| FWER p-Value | 1.0 |
Table: GSEA Results Summary

  

Fig 1: Enrichment plot: GOMF\_S\_ADENOSYL\_L\_METHIONINE\_BINDING      
 Profile of the Running ES Score & Positions of GeneSet Members on the Rank Ordered List

  

| SYMBOL | TITLE | RANK IN GENE LIST | RANK METRIC SCORE | RUNNING ES | CORE ENRICHMENT || 1 | TPMT | thiopurine S-methyltransferase [Source:HGNC Symbol;Acc:HGNC:12014] | 54 | 0.981 | 0.2682 | Yes |
| 2 | N6AMT1 | N-6 adenine-specific DNA methyltransferase 1 [Source:HGNC Symbol;Acc:HGNC:16021] | 330 | 0.734 | 0.4507 | Yes |
| 3 | METTL5 | "methyltransferase 5, N6-adenosine [Source:HGNC Symbol;Acc:HGNC:25006]" | 2107 | 0.349 | 0.4097 | No |
| 4 | ZCCHC4 | zinc finger CCHC-type containing 4 [Source:HGNC Symbol;Acc:HGNC:22917] | 4909 | 0.079 | 0.2144 | No |
| 5 | CBS | cystathionine beta-synthase [Source:HGNC Symbol;Acc:HGNC:1550] | 5285 | 0.049 | 0.1988 | No |
| 6 | PCIF1 | phosphorylated CTD interacting factor 1 [Source:HGNC Symbol;Acc:HGNC:16200] | 5319 | 0.046 | 0.2090 | No |
| 7 | METTL17 | methyltransferase like 17 [Source:HGNC Symbol;Acc:HGNC:19280] | 5673 | 0.018 | 0.1865 | No |
| 8 | KMT5B | lysine methyltransferase 5B [Source:HGNC Symbol;Acc:HGNC:24283] | 6198 | -0.020 | 0.1514 | No |
| 9 | PRMT1 | protein arginine methyltransferase 1 [Source:HGNC Symbol;Acc:HGNC:5187] | 6319 | -0.029 | 0.1502 | No |
| 10 | METTL3 | "methyltransferase 3, N6-adenosine-methyltransferase complex catalytic subunit [Source:HGNC Symbol;Acc:HGNC:17563]" | 6685 | -0.055 | 0.1370 | No |
| 11 | TSR3 | TSR3 ribosome maturation factor [Source:HGNC Symbol;Acc:HGNC:14175] | 7676 | -0.133 | 0.0971 | No |
| 12 | PRMT8 | protein arginine methyltransferase 8 [Source:HGNC Symbol;Acc:HGNC:5188] | 8150 | -0.167 | 0.1067 | No |
| 13 | SUV39H2 | suppressor of variegation 3-9 homolog 2 [Source:HGNC Symbol;Acc:HGNC:17287] | 8529 | -0.196 | 0.1318 | No |
| 14 | GNMT | glycine N-methyltransferase [Source:HGNC Symbol;Acc:HGNC:4415] | 9208 | -0.248 | 0.1482 | No |
| 15 | SETD6 | "SET domain containing 6, protein lysine methyltransferase [Source:HGNC Symbol;Acc:HGNC:26116]" | 11467 | -0.498 | 0.1112 | No |
Table: GSEA details [plain text format]

  

Fig 2: GOMF\_S\_ADENOSYL\_L\_METHIONINE\_BINDING      
 Blue-Pink O' Gram in the Space of the Analyzed GeneSet

  

Fig 3: GOMF\_S\_ADENOSYL\_L\_METHIONINE\_BINDING: Random ES distribution      
 Gene set null distribution of ES for **GOMF\_S\_ADENOSYL\_L\_METHIONINE\_BINDING**
